# Treatment Options That Reduce the Duration of Sickle Cell Vaso-Occlusive Crises: A Systematic Review

**DOI:** 10.7759/cureus.28337

**Published:** 2022-08-24

**Authors:** Adebisi O Akindele, Ana P Jalkh, Aziza K Eastmond, Chaitra Shetty, Syed Muhammad Hannan Ali Rizvi, Joudi Sharaf, Kerry-Ann D Williams, Maha Tariq, Maitri V Acharekar, Sara Elena Guerrero Saldivia, Sumedha N Unnikrishnan, Yeny Y Chavarria, Prachi Balani

**Affiliations:** 1 Research, California Institute of Behavioral Neurosciences & Psychology, Fairfield, USA; 2 Internal Medicine, Saint Vincent Hospital, Worcester, USA

**Keywords:** psychosocial issues sickle cell anaemia, sickle cell children, sickle cell disease (scd), sickle cell trait, vaso-occlusive pain, vaso occlusive crisis, adult sickle cell anemia, haemostasis sickle cell anaemia, painful crisis, sickle cell crisis

## Abstract

Most patients with sickle cell disease (SCD) seek hospital care because of pain symptoms. While some patients opt to treat themselves at home, some prefer to seek treatment in a hospital setting. There are, however, some patients with more complicated effects of the disease who seek treatment so often that they have been termed “super-users.” This paper seeks to determine, across the board, the treatments available for vaso-occlusive crisis (VOC), the most common complication of SCD. Due to the frequency and unpredictable nature of VOC, it is no surprise that the lives of so many patients dealing with SCD are constantly disrupted by this complication. Treatments that reduce the frequency of VOC and the need for hospital admissions will help these patients find some semblance of balance in their quality of life.

## Introduction and background

Around 367 to 500 million people worldwide are carriers of sickle cell disease (SCD) [1]. A world without SCD would mean a world with millions of people not dealing with pain constantly. SCD is one of several hemoglobinopathies that exist. It occurs in an individual who has inherited either one sickle cell gene mutation from both parents or one from one parent and another mutated Hemoglobin (Hb) gene from the other parent [1]. It occurs in both homozygous and heterozygous forms. HbA refers to an unmutated Hb gene, while HbS refers to a gene with the sickle cell mutation. HbAA (HbA + HbA) is found in an individual with both unmutated Hb genes. HbAS (sickle cell trait) is found in someone who inherited just one sickle cell gene. Whereas HbSS (combination of HbS + HbS) represents someone with SCD. These are, however, not the only combinations found in humans. Other Hb mutations are inherited with the sickle cell mutation to create HbSC (HbS + HbC), HbSE (HbS + HbE), and HbSß (HbS + Beta Thalassemia), as explained by Cooper et al. and Khamees et al. [1,2]. Due to all these differences, there are some shared manifestations of SCD, but with different occurrences and severity. Those with HbSS have the most severe forms of signs and symptoms. Contrary to popular belief, symptoms are seen in people with not just the homozygous mutations but with a single Hb sickle cell gene and an accompanying hemoglobinopathy on the other paired gene. Symptoms of SCD typically only begin after about six months of age, when the protective fetal Hb (HbF) is replaced by the adult form. More serious effects of the disease, such as acute chest syndrome, are predominantly reported in patients with the HbSS genotype [2].

The name sickle cell anemia is derived from the shape that the red blood cells (RBCs) take when in the diseased form. The sickled RBCs then accumulate and cause a blockage in blood vessels (vaso-occlusion), causing a variety of symptoms. The sickled RBCs are also broken down at a higher rate, causing an additional type of anemia (hemolytic) [3]. Some of the symptoms are sickle cell pain crisis, acute chest syndrome, leg ulcers, strokes, pulmonary emboli, end-organ damage, infections, and many other problems [1,2,4]. Sickle cell crisis is an acute occurrence, also known as vaso-occlusive crisis (VOC), and this is a very common presenting symptom of sickle cell patients [2]. VOC refers to pain in the bones and joints [1], and episodes could be brought about by dehydration, over-exertion, anemia, cold, and low oxygen levels (e.g., at high altitudes). Due to the acuity of symptoms and the vast comorbidities associated with SCD, it is no surprise that a subset of patients seeks emergent care more often than others [5]. A lot of patients opt out of hospital treatments for reasons unknown. This paper seeks to find out whether emergency or inpatient visits decrease the duration of VOCs. If treating VOCs in outpatient settings produces equivalent recovery times as the former, in what ways can the quality of life of patients with SCD be improved in this regard?

## Review

Methods

The articles used in this systematic review were selected from PubMed, PubMed Central (PMC), and Medline. PMC and Medline are two of the several literature sources that form PubMed. The search strategy included the following keywords: sickle cell anemia, vaso-occlusive, hospitalization, emergency department, and outpatient treatment. Medical Subject Headings (MeSH) searches were performed on the aforementioned keywords and phrases. Each MeSH search was then accompanied by relevant subheadings to yield individual results. 

The subheadings used for each MeSH search are as follows: (1) sickle cell anemia: anemia, sickle cell. Subheadings used are blood, complications, diagnosis, diagnostic imaging, drug therapy, mortality, etiology, prevention and control, therapy, and restrict to MeSH-major topics. (2) Vaso-occlusive. MeSH search produced Sevuparin. Subheadings used are restrict to MeSH-major topics. (3) Hospitalization. Subheadings used are methods, prevention, and control, therapy, numeric and statistical data, restrict to MeSH-major topics, and trends. (4) Emergency department (emergency service, hospital). Subheadings used are methods, standards, statistics, numerical data, and restrict to MeSH-major topics. (5) Outpatient care (ambulatory care). Subheadings used are epidemiology, restrict to MeSH-major topics, mortality, therapeutic use, methods, statistics and numerical data, and therapy.

Table 1 summarizes the search strategy and databases searched.

**Table 1:** Total number of articles from search strategy. MeSH: Medical Subject headings; PMC: PubMed Central.

Search strategy	Database	Number of articles
All five MeSH search results were combined with “AND” or “OR” and searched in PubMed. Vaso-occlusive was searched in PubMed separately, as it didn’t yield any results in the MeSH search.	PubMed, PMC, Medline	2,579

Results

**Figure 1:**
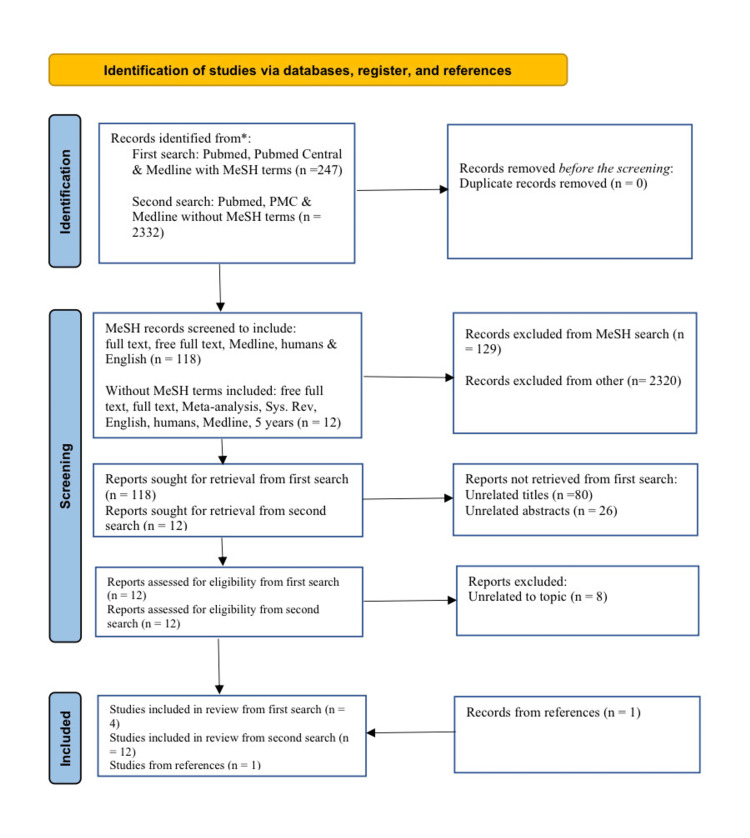
PRISMA flow chart detailing search strategy. MeSH: Medical Subject Headings; PMC: PubMed Central; Sys. Rev: Systematic Review; PRISMA: Preferred Reporting Items for Systematic Reviews and Meta-analyses.

The Preferred Reporting Items for Systematic Reviews and Meta-analyses (PRISMA) [6] guidelines were followed in the final selection of papers for this article. The results of the combined MeSH searches before screening by: full text, free full text, Medline, humans, and English language were 247. The screening then produced 118 results, after which no duplicates were found. A total of 80 were screened out by the title, and 26 were screened out by their abstracts, leaving 12 papers. Eight of these twelve papers were unrelated to the topic, leaving four articles. The results of searching “vaso-occlusive” on its own in PubMed yielded 2,332 papers. These were screened by: free full text, full text, meta-analysis, systematic review, English language, humans, Medline, and five years, to yield another 12 papers. An additional paper was added from references, giving a total of 17 articles. The flow chart demonstrating included and excluded articles is shown in Figure 1.

Discussion

Pathophysiology of VOC

This study is reviewing the treatment options offered in hospital settings. The goal of this paper is to get insight into the options that patients dealing with a VOC have that will improve their quality of life. To understand the various treatment modes currently in use, we will review the mechanisms of pain in VOC. The nature of vaso-occlusive pain is acute and leads to surrounding tissue infarction and inflammation [3,7]. Dehydration is one of the known factors of VOC caused by the formation of deoxyHbS (deoxygenated HbS) which causes increased adherence and reduced flexibility of the RBCs [8-10]. Plasma concentration is a function of the intracellular content of deoxyHbS. The sickling of RBCs is dependent on both of these factors [10]. This is why patients who present with a painful crisis should be given fluids to slow down or stop the sickling process. This can be accomplished by increasing the volume of plasma in the body, which in turn lowers blood viscosity. Indirectly, this also lowers the amount of HbS that is present inside RBCs [10].

The subsequent deoxygenation in the RBCs causes them to take a "sickled" shape. These sickled, damaged cells then attach to the endothelial wall, forming a mass with white blood cells and platelets, with the aid of adhesion molecules (P and E-selectin, intercellular adhesion molecule, and basal cell adhesion molecule-lutheran blood group [BCAM/Lu]) [3,7]. The continued ischemia and increased acidity lead to the breakdown of endothelial cells and RBCs, which, in turn, attract monocytes. Inflammatory molecules comprising tryptase and hypoxia-inducible factor-1 alpha (HIF-1α) are released from infiltrated mast cells to activate nociceptors like transient receptor potential vanilloid type 1 (TRPV1) [3]. The fusion of the sickled RBCs and endothelial cells causes the release of atypical oxygen molecules, which in turn trigger transcription factor nuclear factor (NF-𝜅B). NF-𝜅B furthers the cycle by releasing more adhesion molecules. The aforementioned cycle of events has been proven with sickle cell mouse experiments [7]. This inflammatory response is part of a larger cycle of events that lead to further tissue or organ damage and of course, pain. The molecules released from this process are usually in high amounts, and they activate pain receptors [7]. These pain receptors transfer the stimuli to the spinal cord through the A𝛅 and C fibers. Glutamate is the excitatory molecule that transmits pain, and the inhibitory molecule is gamma-amino-butyrate (GABA) [7]. In defining the nature of VOC, the intensity of the pain input and the type of receptors engaged in processing the stimulus may play a role [7]. 

There may be acute repeated painful crises, persistent pain syndromes, and neuropathic pain associated with nociceptive sickle cell pain. The prodromal, initial, established, and resolving phases are the steps through which the VOC evolves [11]. On the other hand, chronic pain may be caused by avascular necrosis, leg ulcers, or intractable pain in the absence of evident symptoms [11]. Nerve stimuli in intermittent VOC are not very strong and are primarily associated with ɑ-amino-3-hydroxy-5-methyl-4-isoxazole propionic acid. Magnesium typically prevents the opening of N-methyl-D-aspartic acid (NMDA) channels, resulting in only a slight depression of the membrane potential. The NMDA channel is activated as it is subjected to pain stimuli that are both more intense and more frequent. This activation is accompanied by the expulsion of magnesium and the following influx of calcium. Calcium is responsible for activating several different intracellular signaling cascades, which in turn makes it easier for painful sensations to be transmitted [7]. There is a low pain threshold and allodynia in those who have VOC because of a mechanism that is called central sensitization. Central sensitization is a process in which multiple pain signals from the injured area of the body are transmitted to the central nervous system. This results in an extension and intensification of the sensation of pain, even after the damaged tissue has been repaired. SCD animal studies that included imaging abnormalities in the brain and spinal cord lend support to this mechanism [7]. The resulting pain perceived by the patient is from the activating processes listed above [7]. This happens with concurrent pain inhibitory processes via norepinephrine, dynorphin, encephalin, serotonin, and β-endorphin pathways [7].

Chronic pain is typically accompanied by symptoms such as emotional anguish, behavioral dysfunction, tension within the family, concerns regarding one's financial situation, frequent visits to healthcare professionals, extensive usage of analgesic medications, and dread [11]. Table 2 includes a breakdown of the articles that describe sickle cell crises.

**Table 2:** Articles discussing the pathophysiology of VOC. TENS: Transcutaneous electrical nerve stimulation; VOC: Vaso-occlusive crisis.

Study	Author	Year	Type of Study	Patients	Purpose of the Study	Results	Conclusion
1	Takaoka et al. [3]	2021	Traditional Review	0	Understanding the process of pain in Sickle Cell Disease.	Inflammation is the main factor that triggers the pain cycle in VOC.	New pain pathways give hope to the future of VOC treatments.
2	Estcourt et al. [8]	2020	Systematic Review	990	To determine whether or not transfusions are necessary before surgeries to prevent complications in sickle cell patients.	There was no difference in the administration of mild or generous amounts of transfusions. The studies of patients who received and did not receive transfusions showed no difference in complications.	There is not sufficient evidence to support the need for transfusions before surgeries in sickle cell patients.
3	Pal et al. [11]	2020	Systematic Review	22	Evaluating the use of TENS in treating sickle cell pain.	The study was inconclusive, as there are insufficient studies in this area.	It is still unclear whether or not TENS is a potential treatment for VOC.
4	Frimpong et al. [9]	2018	Systematic Review	912	To investigate the potency and side effects of antimalarial drugs in Sickle Cell patients.	Chloroquine, Sulphadoxine-Pyrimethamine, and Pyrimethamine all lowered the frequency of VOC when used individually.	Antimalarial drugs showed some positive effects on VOC.
5	Okomo et al. [10]	2017	Systematic Review	0	To identify the best mode of rehydration during a VOC.	There is a lack of trials measuring the different modes of rehydration in VOC.	Even with the lack of studies to back up the rehydration theory, it is seen as vital in any form of the treatment of VOC.
6	Puri et al. [7]	2017	Traditional Review	0	Overview of the mechanism of pain in VOC, as well as old and new treatments.	The timing, mode, and type of drug administered have different effects on VOC and are factors that should be considered in future studies.	Pain management in VOC is more intricate than we know and will require more rapid ways to perform clinical trials to improve treatment methods.

Available Treatment Options

The most common reason for hospitalization in SCD is VOC [1,10,11]. Treatment for individuals diagnosed with VOC typically begins upon presentation to an emergency department of a healthcare facility, with the requirement of rapid treatment for severe and disabling pain [1]. Some patients decide to treat themselves at home. In either case, the primary goal is to alleviate suffering as quickly as possible by the prompt administration of fluids and medications [1].

People who have SCD almost always have a kidney condition called hyposthenuria, which means they are unable to concentrate their urine. This functional impairment in the concentrating process happens due to hypoxia in the renal tubules and as a consequence of subclinical intravascular sickling in the hypertonic renal medulla. Both of these factors contribute to the renal medulla's hypertonicity [10]. Dehydration caused by undetectable water loss reduced fluid intake, and polyuria induces a reduction in fluid volume along with an increase in blood viscosity. This accelerates and perpetuates the sickling process; hence fluid replacement therapy is of the utmost importance [10].

Opioids and Non-Steroidal Anti-Inflammatory Drugs (NSAIDs)

As a quality care measure for treating patients who have SCD, the initiation of analgesic medication should take place within 30 minutes of the triage process or 60 minutes after registration. During the initial assessment, a pain assessment tool should be employed to identify which class of medication should be administered. Additionally, the precipitating factor that led to the crisis should be evaluated during this phase [7]. As long as it provides the patient some relief, nonsteroidal anti-inflammatory drugs (NSAIDs) are used in the treatment of mild to severe VOC. On the other hand, parenteral opioids should be given if the pain lasts for a longer period or if it is more severe. The choice of opioids should be based on the hematologist’s recommendation (if the patient has one), and the patient's awareness of potentially helpful medications and potentially harmful ones [7]. Studies and clinical trials have shown that patient-controlled analgesia, often known as PCA, as well as periodic appraisal of pain, are beneficial. In the therapy of VOC, supportive care plays an essential role. Because overhydration can lead to acute chest syndrome, considerable attention should be paid to monitoring sedation levels, vital signs, and fluid administration [7]. 

Other treatment options

Various sequelae of SCD can lead to the need for surgery, and transfusions become a necessity in these instances [8]. The simple transfusion is the administration of normal RBCs, while the exchange transfusion involves simultaneous removal of the patient’s blood [8]. To prevent strokes, VOC, and acute chest syndrome, blood transfusions are frequently administered to patients as well [8]. According to the Frimpong et al. study, while antimalarial drugs are recommended by the World Health Organization (WHO), studies have shown that the current recommendations do not improve or prevent VOC in patients in endemic areas [9]. Older, single-drug treatments, such as Sulfadoxine-Pyrimethamine and Chloroquine, did show an effect in decreasing the frequency of VOCs [9]. Malaria parasites, however, are now resistant to these drugs [9]. Hydroxyurea therapy for sickle cell disease has been utilized for many years and appears to be helpful for both the main and secondary prevention of stroke in sickle patients [12]. It does so by elevating the HbF levels that are present in the RBCs of patients. Thus, reducing the number of sickled red blood cells in circulation. Transcutaneous electrical nerve stimulation is the application of electrical stimulation to the skin for pain control, as defined by the American Physical Therapy Association [11]. It is non-invasive, economical, safe, and easy to use; a small device powered by a battery applies an electric current via electrodes that are not intrusive to the skin to activate underlying neurons and, as a result, lessen pain perception. It is possible to use it with a variety of frequencies, ranging from extremely low (10 Hz) to extremely high (> 50 Hz) [11]. Intravenous magnesium sulfate or oral magnesium citrate is another factor that could be used to improve the pain in SCD, as shown in some studies [13]. A few studies have shown that magnesium reduced the duration of VOC [13]. A summary of the articles detailing various treatments is shown in Table 3 below.

**Table 3:** Treatment options for sickle cell crises. TENS: Transcutaneous electrical nerve stimulation. VOC: Vaso-occlusive crisis

Study	Author	Year	Type of Study	Patients	Purpose of the Study	Results	Conclusion
1	Darshana et al. [12]	2021	Systematic Review		To determine the application of Hydroxyurea and blood products in the treatment of sickle cell disease in South Asia.	There is not enough evidence to back up these interventions at the moment.	South Asian sickle cell patients showed an improvement with Hydroxyurea treatments. The various South Asian countries should develop transfusion plans based on their different patient backgrounds.
2	Pal et al. [11]	2020	Systematic Review	22	Evaluating the use of TENS in treating sickle cell pain.	The study was inconclusive, as there are insufficient studies in this area.	It is still unclear whether or not TENS is a potential treatment for VOC.
3	Estcourt et al. [8]	2020	Systematic Review	990	To determine whether or not transfusions are necessary before surgeries to prevent complications in sickle cell patients.	There was no difference in the administration of mild or generous amounts of transfusions. The studies of patients who received and did not receive transfusions showed no difference in complications.	There is not sufficient evidence to support the need for transfusions before surgeries in sickle cell patients.
4	Than et al. [13]	2019	Systematic Review	386	To determine the preventive and curative effects of Magnesium when administered via different routes.	The standard of evidence was poor.	A cohort study did show some efficacy in reducing the number of VOC in patients.
5	Cooper et al. [1]	2019	Randomized Clinical Trial	594	To evaluate the strengths and weaknesses of the drugs used in VOC.	There was insufficient data to produce tangible results.	There are mixed findings about treatment options for sickle cell disease.
6	Frimpong et al. [9]	2018	Systematic Review	912	To investigate the potency and side effects of antimalarial drugs in Sickle Cell patients.	Chloroquine, Sulphadoxine-Pyrimethamine, and Pyrimethamine all lowered the frequency of VOC when used individually.	Antimalarial drugs showed some positive effects on VOC.
7	Okomo et al. [10]	2017	Systematic Review		To identify the best mode of rehydration during a VOC.	There is a lack of trials measuring the different modes of rehydration in VOC.	Even with the lack of studies to back up the rehydration theory, it is seen as vital in any form of the treatment of VOC.
8	Puri et al. [7]	2017	Traditional Review		Overview of the mechanism of pain in VOC, as well as old and new treatments.	The timing, mode, and type of drug administered have different effects on VOC and are factors that should be considered in future studies.	Pain management in VOC is more intricate than we know and will require more rapid ways to perform clinical trials to improve treatment methods.

Sickle Cell Patients Seeking Hospital Care During a Crisis

In the general population of patients, it was found that 52% of patients went to the emergency department because they had received some kind of recommendation to do so from a healthcare professional or friends and family [14]. With sickle cell patients, a hallmark of VOC is that they are frequent and spontaneous, disrupting the quality of life of this subset of patients [15,16]. While some sickle cell patients opt not to seek medical intervention during a VOC, the variety of complications of SCD does make other sickle cell patients more frequent users of inpatient services [15]. According to the findings of some studies, several patients worry about whether or not they are selecting the appropriate level of care and do not want to be labeled as time-wasters [14]. This may be a factor in the reason why not all patients suffering from a VOC seek treatment. More than 50% of emergency department visits are made by only a fifth of sickle cell patients, known as super-users [17]. 

Aside from the risky stem cell transplant procedure and the lack of a definitive cure for SCD, the treatment of the condition is symptomatic in the majority of instances. One of the primary therapeutic goals is to decrease the number of painful crises that patients experience. Hydroxyurea, an anticancer drug, has been the standard and most reliable treatment for this purpose historically [16]. 

Patients diagnosed with SCD may make fewer trips to the emergency room if there is a connection with other outpatient services and/or if those services are used more frequently [18]. According to the findings of one study, individuals with SCD who were seen at an all-encompassing health clinic had a lower frequency of visits to the emergency room, although the reasons for this were not entirely understood. In addition, persons diagnosed with SCD may be able to be diverted away from emergency departments by receiving pain management treatment at SCD day hospitals located in outpatient settings [18]. Some of the determining factors for patients' hospital visits are summarized in Table 4.

**Table 4:** The impact of various treatment settings on the frequency and recovery times in sickle cell crisis. PRO: Patient-Reported Outcome.

Study	Author	Year	Type of Study	Patients	Purpose of the Study	Results	Conclusion
1	Ismail et al. [15]	2020	Cohort	247	To determine the effects of preventive care measures on emergency room visit outcomes.	Despite the low statistical evidence between the number of emergency room visits and preventive care, emergency room visits and lack of preventive care showed a relevant connection.	Consistent preventive care is a factor in how much patients need emergency healthcare services.
2	Sarri et al. [16]	2018	Systematic Review		To determine which Patient-Reported Outcome (PRO) tools are preferable in sickle cell disease.	One’s identity, ego, and coping mechanisms were among the top patient-reported outcomes. Others were their state of mind, ability to operate, religious practices, and family influence.	The tools identified were unable to produce significant results.
3	Simpson et al. [17]	2017	Cohort	10	To determine if care coordination decreases the need for emergency visits.	Creating treatment plans for sickle cell patients reduced the need for immediate readmission and reduced the number of annual visits.	Taking a team approach to treating sickle cell patients who are frequent emergency service utilizers shows a decrease in the need for future emergency services.
4	Coster et al. [14]	2017	Systematic Review		To determine factors that contribute to patients’ choices of healthcare services.	People will generally use emergency services due to the availability of extended working hours.	Most times, people will visit the emergency room because of a lack of primary care services or due to being critically ill, or at the request of loved ones.
5	Yusuf et al. [18]	2010	Traditional Review		To find out data concerning sickle cell patients who visit the emergency room.	Pain was the most common reason for emergency room visits by sickle cell patients, followed by pain in the chest, and then respiratory problems.	Utilization of outpatient treatment options showed a reduced recurrence of emergency room visits.

Limitations

There were not many studies that compared treatments to placebo in reducing the duration of VOC. There was a study that measured the duration of VOC with the administration of magnesium versus placebo. However, there was not enough evidence to support the claim that treatment with magnesium is beneficial.

## Conclusions

Understanding the pathways of pain in VOC is vital in the creation of treatment plans that are less complex than those we have now. There is no one size fits all treatment that is known to shorten vaso-occlusive episodes. Most treatments are individualized based on the level of pain, the hospital procedures, and a previously successful treatment plan. The use of individualized treatment plans developed through a combined multi-disciplinary approach is the most beneficial way to shorten the duration of a crisis. Given that intravenous administration of fluids and treatments are available in the inpatient setting, this would be a preferred method of rehydration. 

It has been shown that there are benefits to working with an interdisciplinary team of specialists, primary care physicians, as well as other mental health professionals. One of the drawbacks that could be the reason for the slow advancement of sickle cell therapy is the cycle of mistrust between patients and physicians in certain regions. Without sufficient randomized clinical trials in sickle cell anemia, there will remain an apparent gap in the management of VOC.

## References

[1] Cooper TE, Hambleton IR, Ballas SK, Wiffen PJ (2016). Pharmacological interventions for painful sickle cell vaso‐occlusive crises in adults. Cochrane Database Syst Rev.

[2] Khamees I, Ata F, Choudry H, Soliman AT, De Sanctis V, Yassin MA (2021). Manifestations of HbSE sickle cell disease: a systematic review. J Transl Med.

[3] Takaoka K, Cyril AC, Jinesh S, Radhakrishnan R (2021). Mechanisms of pain in sickle cell disease. Br J Pain.

[4] Carroll PC, Haywood C Jr, Hoot MR, Lanzkron S (2013). A preliminary study of psychiatric, familial, and medical characteristics of high-utilizing sickle cell disease patients. Clin J Pain.

[5] Carroll CP, Haywood C Jr, Fagan P, Lanzkron S (2009). The course and correlates of high hospital utilization in sickle cell disease: evidence from a large, urban Medicaid managed care organization. Am J Hematol.

[6] Page MJ, McKenzie JE, Bossuyt PM (2021). The PRISMA 2020 statement: an updated guideline for reporting systematic reviews. BMJ.

[7] Puri L, Nottage KA, Hankins JS, Anghelescu DL (2018). State of the art management of acute vaso-occlusive pain in sickle cell disease. Paediatr Drugs.

[8] Estcourt LJ, Kimber C, Trivella M, Doree C, Hopewell S (2020). Preoperative blood transfusions for sickle cell disease. Cochrane Database Syst Rev.

[9] Frimpong A, Thiam LG, Arko-Boham B, Owusu ED, Adjei GO (2018). Safety and effectiveness of antimalarial therapy in sickle cell disease: a systematic review and network meta-analysis. BMC Infect Dis.

[10] Okomo U, Meremikwu MM (2017). Fluid replacement therapy for acute episodes of pain in people with sickle cell disease. Cochrane Database of Systematic Reviews.

[11] Pal S, Dixit R, Moe S (2017). Transcutaneous electrical nerve stimulation (TENS) for pain management in sickle cell disease. Cochrane Database Syst Rev.

[12] Darshana T, Rees D, Premawardhena A (2021). Hydroxyurea and blood transfusion therapy for sickle cell disease in South Asia: inconsistent treatment of a neglected disease. Orphanet J Rare Dis.

[13] Than NN, Soe HH, Palaniappan SK, Abas AB, De Franceschi L (2019). Magnesium for treating sickle cell disease. Cochrane Database Syst Rev.

[14] Coster JE, Turner JK, Bradbury D, Cantrell A (2017). Why do people choose emergency and urgent care services? A rapid review utilizing a systematic literature search and narrative synthesis. Acad Emerg Med.

[15] Ismail AF, Tarawah RA, Azzouni ZY, Alharbi LT, Altayyar RM (2020). The relation between regular outpatient follow-up and frequency of emergency department visits in sickle cell pediatric patients. Saudi Med J.

[16] Sarri G, Bhor M, Abogunrin S, Farmer C, Nandal S, Halloway R, Revicki DA (2018). Systematic literature review and assessment of patient-reported outcome instruments in sickle cell disease. Health Qual Life Outcomes.

[17] Simpson GG, Hahn HR, Powel AA (2017). A patient-centered emergency department management strategy for sickle-cell disease super-utilizers. West J Emerg Med.

[18] Yusuf HR, Atrash HK, Grosse SD, Parker CS, Grant AM (2010). Emergency department visits made by patients with sickle cell disease: a descriptive study, 1999-2007. Am J Prev Med.

